# A Common Cold

**Published:** 1869-03

**Authors:** 


### A COMMON COLD

Is taken by the skin becoming colder than is natural, and the instant a chilly sensation is experienced the mischief has been done; but it can almost always be rectified so that no appreciable ill results will follow, if the person will immediately commence some form of exercise or work and continue it until a gentle perspiration is induced, manifested by a slight moisture on the forehead, if a hat is worn. This condition should be kept up for half an hour, then go at once to a good fire, even if in summer time, and remain ten or fifteen minutes, laying aside the extra garments by degrees; it is greatly better to do this and feel well in an hour or two, than to neglect these precautions in the indefinite hope that no harm will result; but injury will follow, lasting, on an average, about two weeks.

Many persons can tell very decidedly when they have taken a cold, and even then they can ward off its ill results if, beginning within twelve hours after such cold has been taken, nothing whatever is eaten for twenty-four hours.

"Feed a cold and starve a fever" is a great fallacy; we remember at this moment no malady, but madness, which is not alleviated and mainly cured by a judicious starvation. But the first effect of a cold is, if possible, to increase the appetite; this continues for a day or two; hence, not one in a thousand has force of character enough to practice absolute abstinence and thus conquer the disease in a few hours; but in a day or two more, nature takes away the desire for food and the victim proclaims himself "miserable," and remains so for nearly a fortnight longer; one day's voluntary abstinence in the first place would have cured him; but now it is a forced abstinence, not for a day, but for the greater part of ten days, with the discomfort and loss of time thrown in to give good measure.

When a person has let slip the best time for promptly curing a cold and finds himself out of sorts, fit for nothing, and the cold has fairly fastened on him, which is the case in three or four days after it has been taken, the best plan is to go to bed in a warm room with a pure air, and remain in bed until he feels hungry and otherwise comfortable, eating nothing whatever, and drinking nothing but cold water, or, which is better, eat ice to the fullest extent the thirst requires.

Every mouthful of food swallowed after a cold has been fairly taken only makes that much more phlegm in proportion to be " raised " from the lungs, and is the cause of much more cough and fever and discomfort.

The above is the unmedicinal method of treating colds and will always succeed when success is possible. Should these means fail, then consult your family physician, but do not risk a three months' indisposition by consulting a newspaper and buying somebody's " drops " or " candy " or " trochees," for the tendency of all such things is, invariably, to ameliorate the symptoms, but to protract the disease; they all are radically and essentially deceptive.

